# Programmable G-to-Y base editing using engineered DNA glycosylase

**DOI:** 10.1093/lifemedi/lnae005

**Published:** 2024-02-17

**Authors:** Huawei Tong, Tong Li, Hui Yang

**Affiliations:** HuidaGene Therapeutics Co., Ltd., Shanghai 200131, China; HuidaGene Therapeutics Co., Ltd., Shanghai 200131, China; HuidaGene Therapeutics Co., Ltd., Shanghai 200131, China; Institute of Neuroscience, State Key Laboratory of Neuroscience, Key Laboratory of Primate Neurobiology, Center for Excellence in Brain Science and Intelligence Technology, Chinese Academy of Sciences, Shanghai 200031, China

We have developed a deaminase-free glycosylase-based guanine base editor (gGBE) capable of G editing, through the fusion of Cas9 nickase with an engineered N-methylpurine DNA glycosylase protein (MPG). This breakthrough represents a proof-of-concept for a novel base editing approach that empowers the engineered DNA glycosylase with the capability of selectively excising previously unexplored substrate.

**Figure 1.**
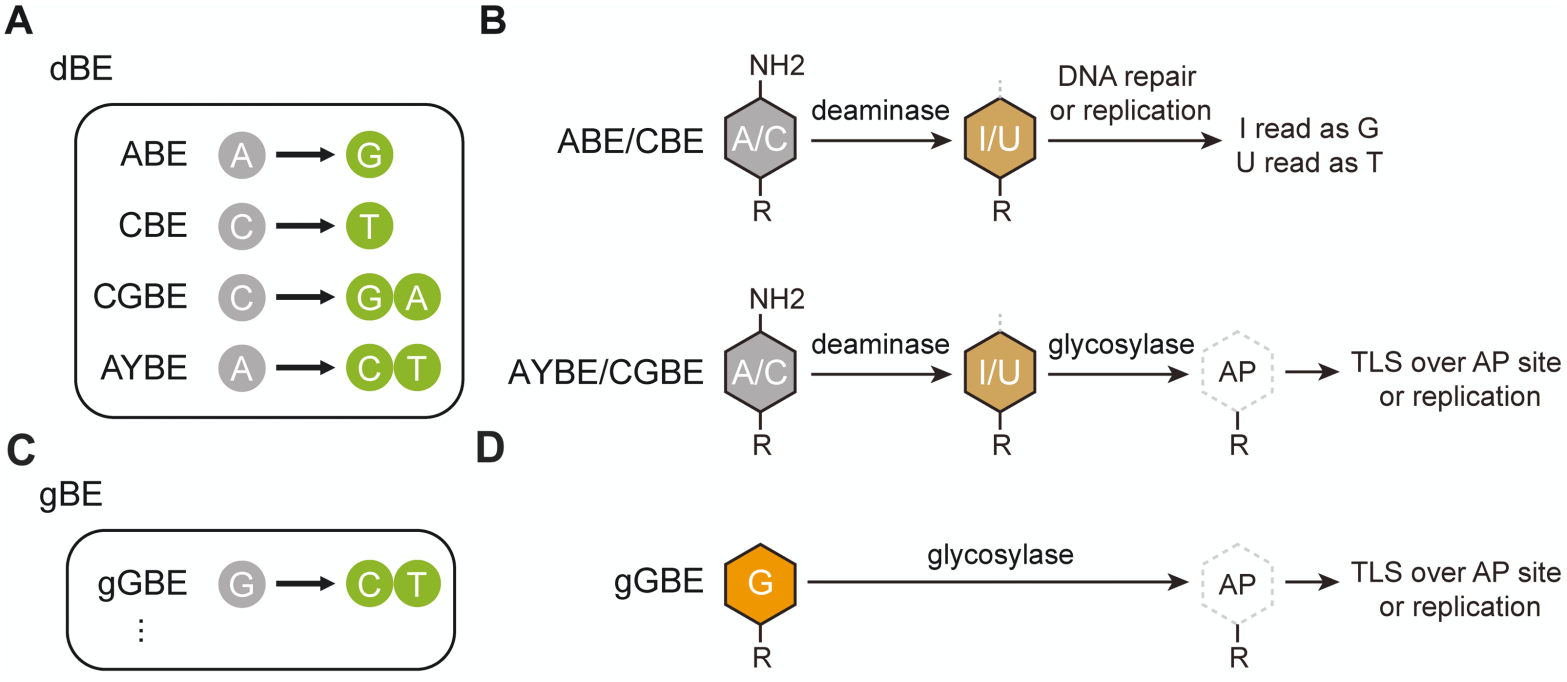
**Outcomes and mechanisms of base editing by deaminase-based and glycosylase-based base editors.** (A) Outcomes of base editing by various dBEs. (B) Schematic diagram of potential pathway for dBEs. In ABE and CBE, the evolved adenine deaminase and AID/APOBEC-like cytidine deaminase perform conversion of the exposed adenine (A) to deoxyinosine (I) and cytosine (C) to deoxyuridine (U), respectively. The DNA polymerase identifies the resultant “I” as “G” and “U” as “T” during DNA repair or replication. In AYBE and CGBE, MPG and Uracil DNA N-glycosylase (UNG) excises the I or U, respectively, which initiates the base excision repair (BER) pathway within cells, resulting in a broader range of possibilities for base editing outcomes. (C) Outcomes of base editing by gBEs. (D) In gGBE, the engineered glycosylase MPG is designed to eliminate G, then the resulting apurinic/apyrimidinic (AP) site generated is mended through translesion synthesis (TLS) and/or DNA replication processes, resulting in a conversion from G to C or G to T.

Many human pathogenic gene mutations are known to be caused by single-base changes. Base editors (BEs) have significantly advanced both basic research and clinical applications by enabling precise editing of specific bases. Currently, there are two widely used types of DNA base editors: adenine base editor (ABE) and cytosine base editor (CBE). They are constructed by fusing programmable DNA-binding proteins, such as Cas9 D10A nickase (nCas9), with either an adenine deaminase or a cytosine deaminase, respectively, to achieve A-to-G and C-to-T base conversions [1, 2]. Recently, researchers have further developed the CGBE base editor, which enables C-to-G conversions in mammalian cells and C-to-A conversions in *Escherichia coli* [3, 4], and the AYBE adenine transversion base editor, which allows A-to-C or A-to-T conversions [5], by fusing CBE or ABE with DNA glycosylases (Fig. 1A). However, so far, all these above base editors initiate the editing process with a deamination reaction of C or A, respectively, producing deoxyuridine (U) or deoxyinosine (I) intermediates, which in turn are transformed into other bases by endogenous DNA repair or replication mechanisms (Fig. 1B). There is no existing base editor capable of directly editing guanine (G). While G in the non-editing strand can be indirectly edited by editing the C in the editing strand, in many cases, this approach is still limited by factors such as the protospacer-adjacent motif (PAM) sequence and editing efficiency, making direct G editing necessary in many situations.

Due to spontaneous repair of deaminated G in DNA, the conventional approach for base editing through deamination of a base could not be directly applied for G editing. To break away from the traditional mindset of deamination-based base editing (dBE), we have come up with a novel concept of utilizing glycosylase to directly excise G, triggering subsequent DNA repair mechanisms and ultimately achieving G editing (Fig. 1C and 1D). Leveraging an engineered glycosylase developed on our HG-PRECISE^®^ platform, we have successfully created a novel base editing tool, namely gGBE, which does not rely on deaminase and achieves high editing efficiency for G-to-C and G-to-T conversions in mammalian cells. Here, we highlight the development and optimization of gGBE, which allows for direct G editing, as well as explore its potential applications.

We first evolved a glycosylase variant with highly efficient G excision activity. Due to the reported low activity of MPG in removing normal G in *in vitro* experiments, we envisioned that excision of canonical G could be achieved by engineering the MPG protein moiety. By fusing MPG at the C-terminus of nCas9, we generated a deaminase-free glycosylase-based base editor (gBE). In our previous study, we have obtained several MPG variants with high efficiency in excising hypoxanthine (Hx) bases [5]. As Hx bears structural similarity to G or A, we assessed the ability of gBEs containing different MPG variants (MPGv0.2 to MPGv3) to excise G or A. To conveniently assess the occurrence of G or A excision events and their respective base editing efficiency, we devised an intron-split EGFP reporter system. In the reporter system, only G-to-T or A-to-T base conversion could correct the mutation in the intron boundary for proper splicing of the EGFP coding sequence, thus activating EGFP expression. We revealed that all the gBEs showed G-to-T but no A-to-T conversion activity. Among them, the gBE containing MPGv3, hereafter termed as gGBEv3, demonstrated the highest G-to-T editing efficiency compared to that of other gGBE variants in cultured human cells. To further enhance the editing efficiency of gGBEv3, we employed structural analysis and rational design of MPG protein, resulting in the formulation of various engineering strategies and the construction of a series of mutant libraries. Through multiple rounds of screening, we eventually obtained gGBEv6.3, which exhibited the highest efficiency for G editing. Compared to gGBEv0.1, which utilizes wild-type MPG and has an editing efficiency of 0.03%, gGBEv6.3 demonstrated a remarkable 1693-fold increase in editing efficiency, representing a significant and substantial improvement in editing efficiency. The editing efficiency of multiple gGBE variants has also been verified at two endogenous genomic sites, showing a significant increase in overall guanine editing efficiency from 6.4% and 7.5% to 78.5% and 80.3%, respectively, while demonstrating a lower frequency of indels.

We also performed a thorough assessment of gGBEv6.3 on 24 target sites within the human genome in HEK293T cells and found a remarkable editing efficiency of up to 81.2% for guanine editing, primarily resulting in G-to-C and G-to-T conversions. G-to-Y edits (where Y = C or T) made up as much as 94% of the edits, and the occurrence of both sgRNA-dependent and sgRNA-independent off-target editing was relatively low. Due to its G-to-Y conversion capability, gGBE enables a range of gene-editing applications, including the editing of splicing sites, introduction of premature termination codons (PTCs), and editing that circumvents PTCs. The gGBEv6.3 effectively edited the splice acceptor site in exon 45 of the human *DMD* (Duchenne muscular dystrophy) gene, resulting in an overall editing efficiency of 30.3%. High editing efficiency of gGBEv6.3 was also observed in mouse cell lines and embryos. In newborn mice, the average efficiency of PTCs introduction in the *Tyr* gene reached 59.4%, peaking at an impressive 94.87%. Additionally, only minimal indels were observed, and over 57% F0 mice exhibited complete *Tyr* gene inactivation or mosaic phenotypes. These findings highlight the significant potential of gGBEv6.3 in various applications, especially in splice site editing and PTCs introduction.

Overall, our study marks the first development of a glycosylase-based guanine base editor, gGBE, which differs from base editors depending on deaminase and showcases efficient G-to-Y editing with relatively low off-target effects. Out of a total of 60,372 human pathogenic single nucleotide polymorphisms (SNPs), approximately 10% are C-to-G and 5% are T-to-G SNPs. While C-to-G SNPs can be corrected by CGBE, the hunt for an efficient sgRNA is often hampered by PAM limitations and a confined editing window. The gGBE could increase the opportunity to find an efficient sgRNA by targeting the opposite strand compared with CGBE. As for T-to-G SNPs, existing base editors struggle to achieve effective G-to-T (or C-to-A on the opposite strand) conversions. The leap forward represented by gGBEv6.3 significantly expands the targeting scope of base editors. Using similar protein engineering approaches, it is also possible to create glycosylase-based base editors tailored for editing specific bases, such as A, C, or T, thereby providing a comprehensive toolkit for gene editing research.
